# Associations Between Motivation and Mental Health in Sport: A Test of the Hierarchical Model of Intrinsic and Extrinsic Motivation

**DOI:** 10.3389/fpsyg.2018.00707

**Published:** 2018-05-08

**Authors:** Rachel B. Sheehan, Matthew P. Herring, Mark J. Campbell

**Affiliations:** ^1^Department of Physical Education and Sport Sciences, University of Limerick, Limerick, Ireland; ^2^Health Research Institute, University of Limerick, Limerick, Ireland

**Keywords:** modeling, motivation, psychology, quantitative study, team sport, depression, self-determination theory

## Abstract

Motivation has been the subject of much research in the sport psychology literature, whereas athlete mental health has received limited attention. Motivational complexities in elite sport are somewhat reflected in the mental health literature, where there is evidence for both protective and risk factors for athletes. Notably, few studies have linked motivation to mental health. Therefore, the key objective of this study was to test four mental health outcomes in the motivational sequence posited by the Hierarchical Model of Intrinsic and Extrinsic Motivation: motivational climate → basic psychological needs → motivation → mental health outcomes. Elite team-sport athletes (140 females, 75 males) completed seven psychometric inventories of motivation-related and mental health variables. Overall, the athletes reported positive motivational patterns, with autonomous motivation and task climate being more prevalent than their less adaptive counterparts. Elevated depressive symptoms and poor sleep quality affected nearly half of the cohort. Structural equation modeling supported pathways between motivational climate, basic needs, motivation, and mood, depressive symptoms, sleep quality, and trait anxiety. Specifically, a task climate was positively associated with the three basic psychological needs, and an ego climate was positively associated with competence. Autonomy and relatedness had positive and negative associations with autonomous and controlled forms of motivation, respectively. Controlled motivation regulations were positively associated with the four mental health outcomes. Integrated regulation had a negative association with anxiety, and intrinsic regulation had a positive association with depressive symptoms. These findings highlight the complexities of and interrelations between motivation and mental health among athletes, and support the importance of considering mental health as an outcome of motivation.

## Introduction

Motivation is a key determinant of behavior in sport. It is a complex construct, with athletes having diverse and dynamic motives for initiating, directing, sustaining, and terminating effort. Athletes can be motivated by internal or external factors, or a combination of both, which may vary by context and time. Due to the longstanding and widespread interest in motivation, researchers have developed theories, evaluated social-environmental factors, identified universal antecedents, and studied other related variables in an effort to understand motivation. The current study contributes to this vast body of work and provides practical applications for elite athletes by encompassing all of these areas within the framework of the Hierarchical Model of Intrinsic and Extrinsic Motivation (HMIEM).

### Self-determination theory

The HMIEM is an extension of Self-Determination Theory (SDT), which addresses the why of behavior, as well as its antecedents and consequences (Deci and Ryan, 2000), and is the most influential theory in competitive sport motivation (Clancy et al., 2016). SDT posits the existence of different motivational types that lie along a continuum from most to least self-determined; that is, from intrinsic motivation to extrinsic motivation to amotivation (Deci and Ryan, 2000). Self-determined motivation involves performing an action out of choice, rather than out of external obligation or internal pressure. Intrinsic motivation is the most self-determined form of motivation, and refers to doing an activity for the pleasure and satisfaction derived from participation. Extrinsic motivation encompasses behaviors that are linked to a separable outcome, and comprises four behavioral regulations: integrated regulation is the most self-determined form of extrinsic motivation, and includes behaviors that are congruent with an individual's self and value system (e.g., basketball player who participates because sport involvement aligns with her values); identified regulation represents actions that are performed out of choice, though they are not attractive in and of themselves (e.g., football player who does strength work because, even though he does not like it, he understands it contributes to his performance); introjected regulation exists when a person internalizes, but does not endorse, external forces (e.g., gymnast who competes to avoid feeling guilty or ashamed); lastly, external regulation refers to behaviors that are regulated by external sources (e.g., swimmer who engages in training in order to get recognition from parents or coaches). Overall, autonomous motivation incorporates actions that athletes undertake volitionally, and, therefore, comprises intrinsic motivation, integrated regulation, and identified regulation. In contrast, controlled motivation involves intra- or inter-personal coercion, and therefore, includes introjected and external regulations. Amotivation lies at the least self-determined end of the motivational continuum, and is defined as a lack of intention of act.

The HMIEM provides a framework for understanding the determinants and consequences of motivation at the global (personality), contextual (life domain), and situational (state) levels (Vallerand, 1997). Specifically, it posits that the degree to which basic psychological needs are satisfied by social-environmental factors influences the degree to which motivation is considered self-determined, which then leads to affective, cognitive, or behavioral consequences (Vallerand, 1997). Of particular interest in the current study is the aforementioned motivational sequence at the contextual level because contextual motivation includes an individual's motivational behavior in a particular life domain (e.g., sport). Overall, substantial cross-sectional (e.g., Standage et al., 2003) and longitudinal (e.g., Gillet et al., 2010) evidence supports the HMIEM.

### Determinants of motivation

Social-environmental factors are collectively called the motivational climate (Ames, 1992), and are innumerable in the sport context (e.g., teammates, sport structures). With that being said, the coach is considered to be one of the most important architects of the motivational climate in sport, such that his/her emphasis on mastery and self-comparison fosters a task motivational climate, and his/her emphasis on outcomes and normative comparison fosters an ego motivational climate (Keegan et al., 2011). This dichotomy emerged from Achievement Goal Theory (Nicholls, 1989), and is the most common conceptualization of motivational climate in sport research (Lindahl et al., 2015). A task climate is associated with adaptive (more positive) outcomes, such as increased competence, intrinsic motivation, and positive affect, whereas an ego climate is associated with maladaptive (more negative) outcomes, such as extrinsic motivation, amotivation, and negative affect (Harwood et al., 2015).

The motivational climate influences motivation through its impact on the basic psychological needs of competence, autonomy, and relatedness (Vallerand, 1997). These needs are “innate psychological nutriments that are essential for ongoing psychological growth, integrity, and well-being” (p. 229; Deci and Ryan, 2000). Competence is the belief that an individual can successfully accomplish a task, autonomy involves freely choosing an action that aligns with an individual's values, and relatedness entails having a connectedness with others. In a task motivational climate, the coach tends to convey trust in athletes' abilities (competence support), offer choices (autonomy support), and consider the athletes' perspectives (relatedness support), which facilitates need satisfaction and leads to self-determined motivation and other adaptive experiences. In an ego motivational climate, however, the coach often uses control and pressure to influence behavior, which do not support basic psychological needs or self-determined motivation. Such a climate has either no association with adaptive outcomes or an association with maladaptive outcomes (Harwood et al., 2015).

### Consequences of motivation

Much evidence points to the associations between motivation and important outcomes across a range of life domains. For example, motivation is related to interest and dropout intentions in education (Gillet et al., 2012), performance and productivity in the workplace (Grant, 2008), and persistence in sport (Sarrazin et al., 2002), with the latter domain being of primary importance in the current investigation. According to SDT, the more self-determined the motivation, the more positive the consequences (Vallerand, 1997). In sport, there is support for the link between self-determined motivation and a range of outcomes, such as objective performance (Gillet et al., 2010), coping (Mouratidis and Michou, 2011), effort (Pope and Wilson, 2012), decreased burnout (Isoard-Gautheur et al., 2012), enjoyment (Rottensteiner et al., 2015), and mental health (Stenling et al., 2015). Associations between motivation and mental health are supported by early work in SDT (Deci and Ryan, 2008), and continue to be of interest in sport (e.g., Sheehan et al., in press).

There has been a recent upsurge in mainstream interest in athlete mental health, particularly at the elite level (Uphill et al., 2016). Mental health is a “state of well-being in which an individual can realize his or her own potential…” (World Health Organisation, 2016), which comprises numerous variables such as mood and sleep quality (Sheehan et al., in press). When such variables are undermined, an athlete may experience poor mental health in the form of depressed mood and disturbed sleep, as examples. On the one hand, elite athletes occupy a privileged position in society and, arguably, experience heightened pressure on and off the field. On the other hand, however, they may also be more resilient to poor mental health due to the coping strategies honed by their demanding lifestyles. Either way, sport involvement does not imply immunity from poor mental health, and SDT may provide insights into this important outcome. Associations between motivation and mental health have been found in studies that consider the full sequence (e.g., Stenling et al., 2015) or part of the sequence (e.g., Stenling et al., 2016) posited by the HMIEM. In some investigations, mental health is conceptualized as a single outcome (e.g., psychological well-being, Stenling et al., 2015), whereas in others separate components are prioritized according to the specific research question (e.g., exhaustion, Adie et al., 2008). Overall, the evidence suggests that mental health can play a role in the aforementioned four-part motivational sequence, and that assessing numerous mental health variables, as in the current study, may provide greater insight than assessing them in isolation or conceptualizing them as a single outcome.

### Motivation and mental health

The literature indicates that anxiety, poor mood, depression, and disturbed sleep persist in elite sport, and are of interest to most stakeholders because they can impair performance. Notably, these mental health issues have also been linked to elements of the HMIEM (e.g., motivational climate, Abrahamsen and Pensgaard, 2012). Woodman and Hardy (2003) reported a significant negative association between anxiety and competitive performance, while Abrahamsen and Pensgaard (2012) found that decreasing perceptions of a task motivational climate were related to more performance worries. A substantial body of evidence points to the relationship between mood and recovery/training load (e.g., Morgan et al., 1987), which is a central consideration in elite sport due to the extreme physical demands it imposes. Notably, motivation has been found to be a possible contributor to this relationship (Raglin et al., 1990). The elite sport environment contains numerous stressors and constraints that may contribute to depression and, therefore, undermine performance (Doherty et al., 2016). Furthermore, associations between motivation and depression have been previously reported (e.g., Wang et al., 2017), and Stenling et al. (2016) specifically measured depression when examining part of the HMIEM. Sleep disturbance is very common among athletes, can affect training and competition (Gupta et al., 2016), and has recently been investigated with motivation among elite athletes (Sheehan et al., in press). The HMIEM is a potentially useful framework for understanding anxiety, mood, depression, and sleep quality, which are variables of interest in elite sport because they are associated with performance. Overall, concurrent examination of motivation and mental health will contribute to existing theory, while also providing suggestions to improve motivation, mental health, and performance.

### The current study

Against this backdrop, the key objective of this study was to use structural equation modeling (SEM) to integrate four mental health outcomes into the motivational sequence posited by the HMIEM: motivational climate → basic psychological needs → motivation → mental health outcomes. It was hypothesized that: (1) a task motivational climate would be positively associated with basic psychological needs; (2) an ego motivational climate would have no association or a negative association with basic psychological needs; (3) basic psychological needs would be positively associated with autonomous forms of motivation, and negatively associated with controlled forms of motivation; (4) controlled forms of motivation would be positively associated with mental health outcomes; and (5) autonomous forms of motivation would be negatively associated with mental health outcomes.

## Methods

### Participants

Team-sport athletes (*N* = 215; 65% female, 35% male) playing at the highest national level within their sport were recruited. They represented 11 teams across six sports (basketball, Gaelic football, hockey, hurling, rugby, and soccer), ranged in age from 18 to 37 (*M* = 22.8, *SD* = 4.1) years, and had over 12 years sport experience on average (*M* = 12.5, *SD* = 5.0). According to the eliteness classification system (Swann et al., 2014), which encompass athletes' highest standard of performance, their success at that level, the amount of experience that they have gained at that level, and the national competitiveness of the sport, three teams were categorized as “competitive-elite,” and eight teams were categorized as “semi-elite” (Table 1). Eight teams contained athletes who would be classified as “successful elite” or “world-class elite” in their sport; however, the classification system was applied to full teams, rather than individual athletes, in order to capture the level of the team-sport sample.

**Table 1:** Eliteness calculations for athletes according to Swann et al. (2014) classification system.

**Team**	**A**	**B**	**C**	**D**	**E**	^*^Score	**Category**
Women's soccer	3	2	2	3	4	4.08	Competitive-elite
Women's rugby	3	2	2	3	4	4.08	
Men's soccer	3	2	2	3	4	4.08	
Women's soccer	3	2	1	3	4	3.50	Semi-elite
Women's basketball	1	2	2	1	4	2.08	
Women's hockey	1	2	2	1	4	2.08	
Women's Gaelic football	2	2	2	3	1	2.00	
Women's basketball	1	1	1	1	4	1.25	
Men's rugby	2	2	1	3	4	2.92	
Men's hockey	1	2	2	1	4	2.08	
Men's hurling	2	1	2	3	1	1.67	

A, athlete's highest standard of performance; B, success at athlete's highest level; C, experience at athlete's highest level; D, competitiveness of sport in athlete's country; E, global competitiveness of sport;

* *eliteness score = [(A+B+C/2)/3] X [(D+E)/2]*.

### Procedure

The protocol was approved by the University Ethics Committee, and all participants provided written informed consent prior to participation. Seven psychometric inventories were then administered online the following week using Survey Monkey (https://www.surveymonkey.com/). Inventories were selected based on their suitability for the study's objectives, and their well-established psychometric properties (Clancy et al., 2017). Data collection took place between April and November 2015.

### Measures

#### Sport motivation scale II

Motivation was measured using the 18-item Sport Motivation Scale II (SMS-II; Pelletier et al., 2013). The SMS-II asks athletes “why do you practice your sport?” and provides a seven-point Likert scale for each response. It provides scores for intrinsic, integrated, identified, introjected, external, and non regulations. The SMS-II has Cronbach's alpha values ranging from 0.73 to 0.86 for the six subscales (Pelletier et al., 2013).

#### Basic need satisfaction in sport scale

Athletes' perceptions of their competence, autonomy, and relatedness were measured using the Basic Need Satisfaction in Sport Scale (BNSSS; Ng et al., 2011). This 20-item scale asks athletes how they feel when participating in their sport, and provides a seven-point Likert scale for each response. The BNSSS has demonstrated acceptable reliability (Cronbach's alpha of 0.61–0.82 for the five subscales; Ng et al., 2011).

#### Perceived motivational climate in sport questionnaire II

Athletes' perception of the motivational climate typically experienced on their teams was assessed using the 33-item Perceived Motivational Climate in Sport Questionnaire II (PMCSQ-II; Newton et al., 2000). The PMCSQ-II uses the stem “On this team…” and provides scores for perceived task and ego climates. Responses are indicated on a five-point Likert scale. Cronbach's alpha coefficients of 0.88 and 0.87 have been reported for task and ego climates, respectively (Newton et al., 2000).

#### Profile of mood states— brief

Total mood disturbance (TMD) was measured using the Profile of Mood States-Brief (POMS-B; McNair et al., 1971). The POMS-B consists of 30 adjectives describing how the respondent may be feeling right at this moment for five negative mood states (tension, depression, anger, fatigue, and confusion) and one positive mood state (vigor). TMD is calculated by subtracting the total for the vigor items from the total for the negative mood states items, with higher scores indicating greater mood disturbance (range of −20 to 100). The subscales of the POMS-B have acceptable internal consistency of between 0.71 and 0.88 (Yeun and Shin-Park, 2006).

#### Quick inventory of depressive symptomatology—self report

Depressive symptom severity was assessed using the 16-item Quick Inventory of Depressive Symptomatology–Self Report (QIDS-SR; Rush et al., 2003). The QIDS-SR measures nine symptom domains according to the Diagnostic and Statistical Manual of Mental Disorders (American Psychiatric Association, 2013). Respondents are asked to rate symptoms (sad mood, concentration, self-criticism, suicidal ideation, interest, energy/fatigue, sleep disturbance, appetite/weight, and psychomotor agitation/retardation) from the prior 7 days, with the following depressive symptoms classifications: none (0–5), mild (6–10), moderate (11–15), severe (16–20), and very severe (21–27). High internal consistency (α = 0.86) has been reported for the QIDS-SR (Rush et al., 2003).

#### Pittsburgh sleep quality index

Sleep quality was measured using the 19-item Pittsburgh Sleep Quality Index (PSQI; Buysse et al., 1989). The PSQI generates seven component scores that quantify overall sleep quality for the preceding month (good quality = 0–5; poor quality = >5): subjective sleep quality, sleep latency, sleep duration, habitual sleep efficiency, sleep disturbances, use of sleeping medication, and daytime dysfunction. The component scores of the PSQI have a reliability coefficient of 0.83.(Buysse et al., 1989).

#### State-trait anxiety inventory—Y2

Trait anxiety was measured using the State-Trait Anxiety Inventory–Y2 (STAI-Y2; Spielberger et al., 1983). Athletes rated how they generally feel on a four-point Likert scale in response to 20 items. Categorization based on age-related norms approximates high trait anxious individuals as having a score of 50 or higher (range of 20–80; Spielberger et al., 1983). A Cronbach's alpha value of 0.90 has been reported for the STAI-Y2 (Spielberger et al., 1983).

### Data analyses

SPSS Statistics 21.0 and MPlus Version 7.4 were used for the analyses. Athletes' scores for the inventories were summarized using descriptive statistics, and Pearson's correlation coefficients were used to quantify associations between variables (0.10 is small, 0.30 is moderate, and 0.50 is large; Cohen, 1988). Cronbach's alpha coefficients were calculated to examine internal consistency, with 0.70 being the acceptable cut-off (Nunnally, 1978). SEM, specifically a full latent variable model, was used to examine relationships between latent variables. To evaluate the fit of the SEM model, the Tucker-Lewis Index (TLI; >0.90), Comparative Fit Index (CFI; >0.90), Root Mean Square Error of Approximation (RMSEA; <0.10), and Standardized Root Mean Squared Residual (SRMR; <0.08) scores were calculated (Hu and Bentler, 1998). In further examining the SEM, beta (β; standardized regression coefficient) was used to quantify the relationship between variables (0.10 is small, 0.30 is moderate, and 0.50 is large).

## Results

### Preliminary analyses

Descriptive statistics and correlations are presented in Table 2. On average, the athletes had low (good) TMD and trait anxiety, exhibited no depressive symptoms (although they were approaching the cut-score for mild depressive symptoms), and were poor sleepers. When categorized as individuals (using the cut-scores for each inventory), 45% had mild-to-moderate depressive symptoms (46% females, 43% males), 42% were poor sleepers (42% females, 41% males), and 13% were high trait anxious (10% females, 17% males).

**Table 2:** Descriptive statistics and correlations for study variables.

**Variable**	***M***	***SD***	**1**	**2**	**3**	**4**	**5**	**6**	**9**	**10**	**11**	**12**	**13**	**14**	**15**	**16**
IR	4.93	1.40														
InteR	4.81	1.38	0.658^**^													
IdR	4.76	1.48	0.746^**^	0.760^**^												
IntrR	3.76	1.30	0.471^**^	0.554^**^	0.551^**^											
ExR	2.21	1.18	0.149^*^	0.198^**^	0.216^**^	0.417^**^										
NR	1.60	0.89	−0.344^**^	−0.312^**^	−0.241^**^	−0.021	0.292^**^									
Com	5.38	1.10	0.357^**^	0.408^**^	0.358^**^	0.141^*^	−0.031	−0.314^**^								
Aut	5.38	0.93	0.453^**^	0.470^**^	0.391^**^	0.141^*^	−0.131	−0.414^**^	0.714^**^							
Rel	5.57	1.05	0.287^**^	0.301^**^	0.243^**^	0.206^**^	−0.006	−0.164^*^	0.477^**^	0.492^**^						
TC	4.15	0.46	0.409^**^	0.332^**^	0.336^**^	0.173^*^	−0.057	−0.207^**^	0.464^**^	0.564^**^	0.461^**^					
EC	2.44	0.63	−0.153^*^	−0.039	−0.078	0.017	0.175^**^	0.201^**^	−0.088	−0.314^**^	−0.156^*^	−0.494^**^				
TMD	9.90	12.39	−0.036	0.002	0.009	0.214^**^	0.263^**^	0.316^**^	−0.092	−0.118	−0.086	−0.131	0.086			
Dep	5.33	3.00	−0.034	−0.123	−0.018	0.062	0.146^*^	0.395^**^	−0.189^**^	−0.204^**^	−0.165^*^	−0.103	0.174^*^	0.454^**^		
SlQ	5.33	2.40	0.075	0.026	0.097	0.178^**^	0.199^**^	0.243^**^	−0.096	−0.058	−0.052	−0.111	0.151^*^	0.445^**^	0.559^**^	
Anx	39.31	8.62	−0.129	−0.184^**^	−0.057	0.091	0.276^**^	0.435^**^	−0.336^**^	−0.288^**^	−0.244^**^	−0.242^**^	0.183^**^	0.426^**^	0.580^**^	0.438^**^

** *p < 0.01*,

* *p < 0.05, M, mean, SD, standard deviation; IR, intrinsic regulation; InteR, integrated regulation; IdR, identified regulation; IntrR, introjected regulation; ExR, external regulation; NR, non-regulation; Com, competence, Aut, autonomy, Rel, relatedness; T/E C, task/ego climate; TMD, total mood disturbance; Dep, depressive symptoms; SlQ, sleep quality; Anx, trait anxiety*.

### Reliability of the scales

Cronbach's alpha coefficient was 0.61–0.85 for the SMS-II subscales, with values for introjected (α = 0.61), external (α = 0.68), and non (α = 0.68) regulations falling just below the acceptable 0.70 cut-off (Nunnally, 1978). Given that these subscales had demonstrated adequate reliability in previous work (e.g., Pelletier et al., 2013), contained few items, and would not have had an increased alpha coefficient if any item was deleted, they were retained for further analyses. Task (α = 0.90) and ego (α = 0.89) climates, competence (α = 0.89), autonomy (α = 0.86), relatedness (α = 0.83), STAI-Y2 (α = 0.89), and POMS-B (α = 0.87) had acceptable reliability. Although the QIDS-SR (α = 0.58) and PSQI (α = 0.57) did not reach the 0.70 criterion (Nunnally, 1978), both have been widely used and demonstrated strong psychometric properties in sport research (Currie and Johnston, 2016; Gupta et al., 2016).

### Structural equation modeling

**Figure 1:**
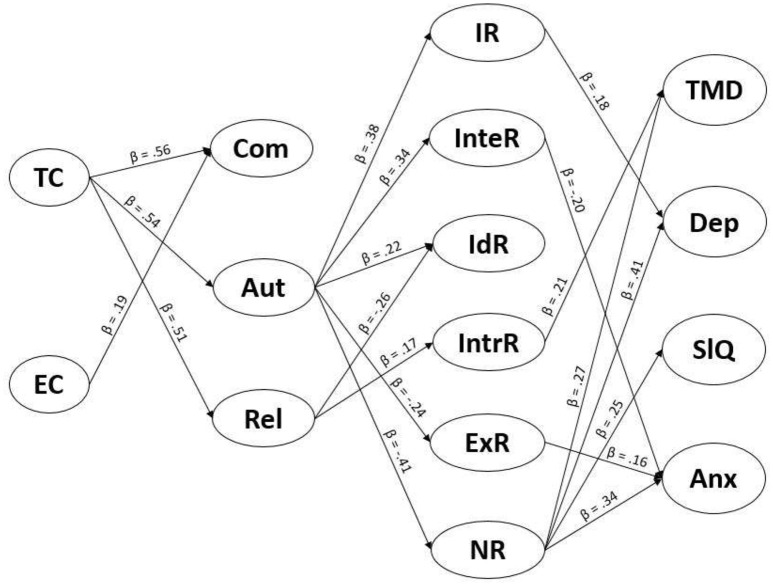
SDT-based model of motivational climate, basic psychological needs, motivation, and four mental health outcomes. Only significant pathways shown. β represents the size and direction of associations between variables. T/E C, task/ego climate; Com, competence, Aut, autonomy, Rel, relatedness; IR, intrinsic regulation; InteR, integrated regulation; IdR, identified regulation; IntrR, introjected regulation; ExR, external regulation; NR, non regulation; TMD, total mood disturbance; Dep, depressive symptoms; SlQ, sleep quality; Anx, trait anxiety.

In support of the key objective of this study, the SDT-based model including mental health outcomes showed acceptable fit to the data (Figure 1): $\chi_{\left(32\right)}^{2}$ = 62.01, *p* < 0.01, CFI = 0.97, TLI = 0.91, RMSEA = 0.07, 90% confidence interval [0.04, 0.09], SRMR = 0.04.

#### Hypothesis 1

In support of hypothesis 1, task climate had a large positive association with competence (β = 0.56), autonomy (β = 0.54), and relatedness (β = 0.51).

#### Hypothesis 2

In partial support of hypothesis 2, ego climate was not associated with autonomy or relatedness, but had a small positive association with competence (β = 0.19).

#### Hypothesis 3

In partial support of hypothesis 3, autonomy had a small-to-moderate positive association with intrinsic (β = 0.38), integrated (β = 0.34), and identified (β = 0.22) regulations, and a small-to-moderate negative association with external (β = −0.24) and non (β = −0.41) regulations. It was not significantly associated with introjected regulation. Furthermore, relatedness had a small negative association with identified regulation (β = −0.26), and a small positive association with introjected regulation (β = 0.17). Lastly, there were no significant associations between competence and any motivation regulation.

#### Hypothesis 4

In support of hypothesis 4, there were small-to-moderate positive associations between introjected regulation and TMD (β = 0.21), external regulation and anxiety (β = 0.16), and non-regulation and each mental health outcome (TMD, β = 0.27; depressive symptoms, β = 0.41; sleep quality, β = 0.25; anxiety, β = 0.34).

#### Hypothesis 5

In partial support of hypothesis 5, there was a small negative association between integrated regulation and anxiety (β = −0.20), but also a small positive association between intrinsic regulation and depressive symptoms (β = 0.18). Identified regulation was not significantly associated with any mental health outcome.

## Discussion

Grounded in SDT and extending previous motivational research, the key objective of this study was to test a model with hypothesized relationships between motivational climate, basic psychological needs, motivation, and four mental health outcomes. The present findings support this model, in which TMD, depressive symptoms, sleep quality, and trait anxiety comprise part of the motivational sequence posited by the HMIEM. Furthermore, the hypothesized associations in the model were partially supported. Overall, the athletes reported positive motivational patterns, with autonomous motivation and task climate being more prevalent than their less adaptive counterparts, and high (above the midpoint) basic needs satisfaction. From a mental health perspective, depressive symptoms and poor sleep quality were reported by nearly half of the cohort. The findings fill a gap in the literature by revealing relationships between traditional elements of the HMIEM and novel outcome variables. Importantly, the study may also help to solve real-world problems for athletes experiencing poor motivation, mental health, or performance.

### Motivational climate and basic psychological needs

The current results indicate that motivational climate had significant associations with basic psychological needs. A task climate had positive relationships with competence, autonomy, and relatedness, which is consistent with previous research on its adaptive effects (Reinboth and Duda, 2006). That is, a coach who endorses a task climate will instill belief in, support free will of, and empathize with athletes, which leads to basic needs satisfaction. The finding that an ego climate did not have any association with autonomy or relatedness is similar to previous reports in which an ego climate was not linked with basic needs satisfaction (Standage et al., 2003), though others have reported a negative association between an ego climate and basic needs satisfaction (Sarrazin et al., 2001). To that end, the positive association between ego climate and competence was unexpected. Although there is no mention of teammates/competitors in the BNSSS, it is possible that an athlete in an ego climate would consider other individuals when interpreting competence items because normative-referenced standards supersede self-referenced standards. Thus, such an athlete could potentially interpret “I am skilled at my sport” as “I am more skilled at my sport than others.” With this in mind, normative-referenced competence may be enhanced in an ego climate when an athlete outperforms competitors (Harwood et al., 2015), which would account for the positive association between ego climate and competence in the current model.

### Basic psychological needs and motivation

Basic needs were significantly associated with some, but not all, motivational regulations. Firstly, autonomy was significantly associated with five of the six motivational regulations. Specifically, the magnitude and direction of these associations were in line with the self-determination continuum, such that they progressively decreased from intrinsic regulation to non-regulation, and turned from positive to negative as there was a shift from autonomous to controlled motivation. The influence of autonomy on autonomous motivation reinforces original work on basic needs, as does the inverse relationship between autonomy and controlled motivation (Deci and Ryan, 2000). Secondly, relatedness was negatively associated with the least self-determined autonomous motivation (identified regulation). Although this is counter to theoretical predictions that relatedness enhances autonomous motivation, it may be attributed to the fact that relatedness emphasizes a connectedness with others, whereas identified regulation is underpinned by personal importance. That is, there may be very minor discord between this somewhat social need and the internalization of behavior typified by identified regulation (notably, the association was small). Thirdly, relatedness was positively associated with the most self-determined controlled motivation (introjected regulation). Although introjected regulation is a controlled form of motivation, it can comprise behaviors that are performed to obtain social recognition. Following this, therefore, an athlete is more likely to feel obligated to do something (introjected regulation) when he/she feels connected with others (relatedness), as in team sport. Fourthly, the lack of an association between competence and motivation was counter to the current hypotheses and much prior evidence (e.g., Chatzisarantis et al., 2003). The aforementioned association between an ego climate and competence may offer an explanation, such that the athletes' perception of competence may be normative-referenced, thereby potentially undermining expected associations between this basic need and self-determination.

### Motivation and mental health

The hypothesized relationships between motivation regulations and mental health outcomes were partially supported. Thus, the findings simultaneously extend theoretical knowledge and provide potential avenues for improving these variables among athletes. The three controlled forms of motivation were positively associated with mental health outcomes, reinforcing longstanding research of the maladaptive effects of such motivation (Deci and Ryan, 2008). It is unsurprising that prioritizing external standards of self-worth and social approval (introjected regulation) likely leads to suboptimal outcomes, such as poor mood, because such standards are analogous to coercion or pressure. Similarly, athletes with external contingencies of reward or punishment (external regulation) may experience increased anxiety because are vulnerable to the perceived judgements of others, which are beyond their control. The finding that non regulation had a positive relationship with the four mental outcomes is in line with the tenets of SDT (Deci and Ryan, 2008). When athletes lack intentionality with their behavior, there are maladaptive consequences, such as psychological ill-being (Vallerand, 1997). In similar studies, non-regulation was found to be associated with low self-esteem, social physique anxiety, and body dissatisfaction in dancers (Quested and Duda, 2011), and with perceived stress in coaches (Alcaraz et al., 2015). This indicates that a complete lack of self-determination not only undermines behavior on the field of play, but may increase mood disturbance, depressive symptoms, and trait anxiety. The association between non regulation and poor sleep quality has been somewhat alluded to in research on athlete burnout, in which amotivation was found to increase exhaustion (e.g., Lonsdale and Hodge, 2011), though it is possible that exhaustion would improve sleep quality.

Turning to autonomous motivation, the negative association between integrated regulation and trait anxiety echoes SDT research, in that increased self-determination tends to produce adaptive outcomes (Deci and Ryan, 2008). Much evidence supports that autonomous motivation contributes to psychological well-being either by enhancing positive mood states or diminishing negative mood states. With this in mind, the positive relationship between intrinsic regulation and depressive symptoms is counter to hypotheses and previous research. As examples, autonomous motivation has been shown to reinforce positive mental health outcomes in exercise (Rouse et al., 2011) and education (Huang et al., 2016) settings. However, such was not the case among the current athletes, in that intrinsic regulation, the most autonomous form of motivation, had a positive association with depressive symptoms. Given that depression has become more prevalent in modern sport due to its increasing physical and psychological demands (Newman et al., 2016), perhaps athletes typified by intrinsic regulation may still experience symptoms of depression. Overall, however, the majority of associations between motivation and mental health outcomes in the current study were consistent with past research and theory.

### Implications

To the authors' knowledge, this is the first study to examine associations between motivation and four mental health outcomes in the HMIEM. Therefore, it makes a significant contribution to a well-established area in the literature. In addition to motivation being essential for optimal performance, this study provides supportive evidence for the link between motivation and mental health. Given the constant emphasis on performance and emerging emphasis on mental health in modern sport, the current findings provide particularly important implications within and beyond the playing field. Evidently, it is important for coaches, parents, et cetera to consider the athletes' motivation when assessing their risk for poor mental health. Moreover, understanding athletes' mental health may better elucidate the underpinnings of their motivation. The significant associations between controlled forms of motivation, and elevated depressive and anxiety symptoms and poor sleep quality and mood provide further evidence of the maladaptive influence of non-self-determined motivation. Therefore, given the links between motivational climate and motivation, coach-centered interventions could be fruitful avenues for positive change for athlete motivation and mental health. Additionally, educational workshops targeting athletes could be beneficial to generate greater understanding in this area. The results could also contribute to athlete development and high-performance strategies devised by governing bodies. For example, psychological monitoring could be made a compulsory component for national teams that meet for irregular and discrete blocks of time (e.g., training camps, tournaments), but otherwise spend extensive and unsupervised periods outside the team environment.

### Limitations

Although this study contributes to the motivation literature by incorporating aspects of mental health, it has several limitations. Firstly, causality cannot be addressed because of the lack of temporal sequence resulting from its cross-sectional design. Experimental or longitudinal designs would allow causal inferences to be made, and are, therefore, warranted for future research. Secondly, only one mental health outcome exceeded its cut-score for the cohort, suggesting that it was a predominantly healthy cohort. Therefore, future research with athletes who have a clinical mental health diagnosis may reveal different relationships to those presented in the SEM (e.g., larger beta values between controlled forms of motivation and mental health outcomes). Thirdly, measurement of psychological needs thwarting would be useful given its association with controlled forms of motivation (e.g., Healy et al., 2014) and negative outcomes (e.g., Bartholomew et al., 2011). Fourthly, the size, level, location, and structure of the sample somewhat limit the generalizability of the findings. Replicating the study with other athlete populations is, therefore, warranted. Fifthly, examination of gender and sport effects, and the interaction of these variables, was not possible due to sample size and composition. Finally, self-report measures may be subject to bias, and could be augmented with other methods (e.g., interviews) in order to provide greater depth of information.

## Conclusion

This is the first study to concurrently investigate four mental health outcomes within the HMIEM among elite athletes, thereby addressing a gap in the literature and providing novel practical implications. Though the cohort displayed predominantly positive motivational patterns, the mental health results were less adaptive. That is, the potentially distress-buffering effects of athletic-involvement were not conclusively supported. In terms of the study's key objective, a model showing relationships between motivational climate, basic psychological needs, motivation, and TMD, depressive symptoms, sleep quality, and anxiety was supported. Thus, this study supports and extends previous research in sport psychology. Specifically, a task climate was positively associated with the three basic psychological needs, and an ego climate was positively associated with competence. Autonomy and relatedness had positive and negative associations with autonomous and controlled forms of motivation, respectively, while competence was unrelated to any motivation regulation. Controlled motivation regulations were positively associated with the four mental health outcomes, with non-regulation having the largest effect. Integrated regulation had a negative association with anxiety, but intrinsic regulation had a positive association with depressive symptoms. These findings underscore the complexity of motivation and mental health in sport, and support the importance of considering mental health as an outcome of motivation.

## Ethics statement

This study was carried out in accordance with the recommendations for human subjects research from the Faculty of Education and Health Sciences Research Ethics Committee. The protocol was approved by the Faculty of Education and Health Sciences Research Ethics Committee at the University of Limerick (2014_12_31). All subjects gave written informed consent in accordance with the Declaration of Helsinki.

## Author contributions

RS, MH and MC have satisfied all the criteria for authorship: Substantially contributing to the study's conception, and interpretation; drafting and revising the work; approving the version to be published; agreeing to be accountable for the work.

### Conflict of interest statement

The authors declare that the research was conducted in the absence of any commercial or financial relationships that could be construed as a potential conflict of interest.
